# Supplementary material for: Structural features of Dnase1L3 responsible for serum antigen clearance
Source: Commun Biol. 2022 Aug 16;5:825. doi: 10.1038/s42003-022-03755-5 (PMC9381713; doi:10.1038/s42003-022-03755-5)
Supplement: Supplementary file 2 — Supplemental Material [file 42003_2022_3755_MOESM2_ESM.pdf]

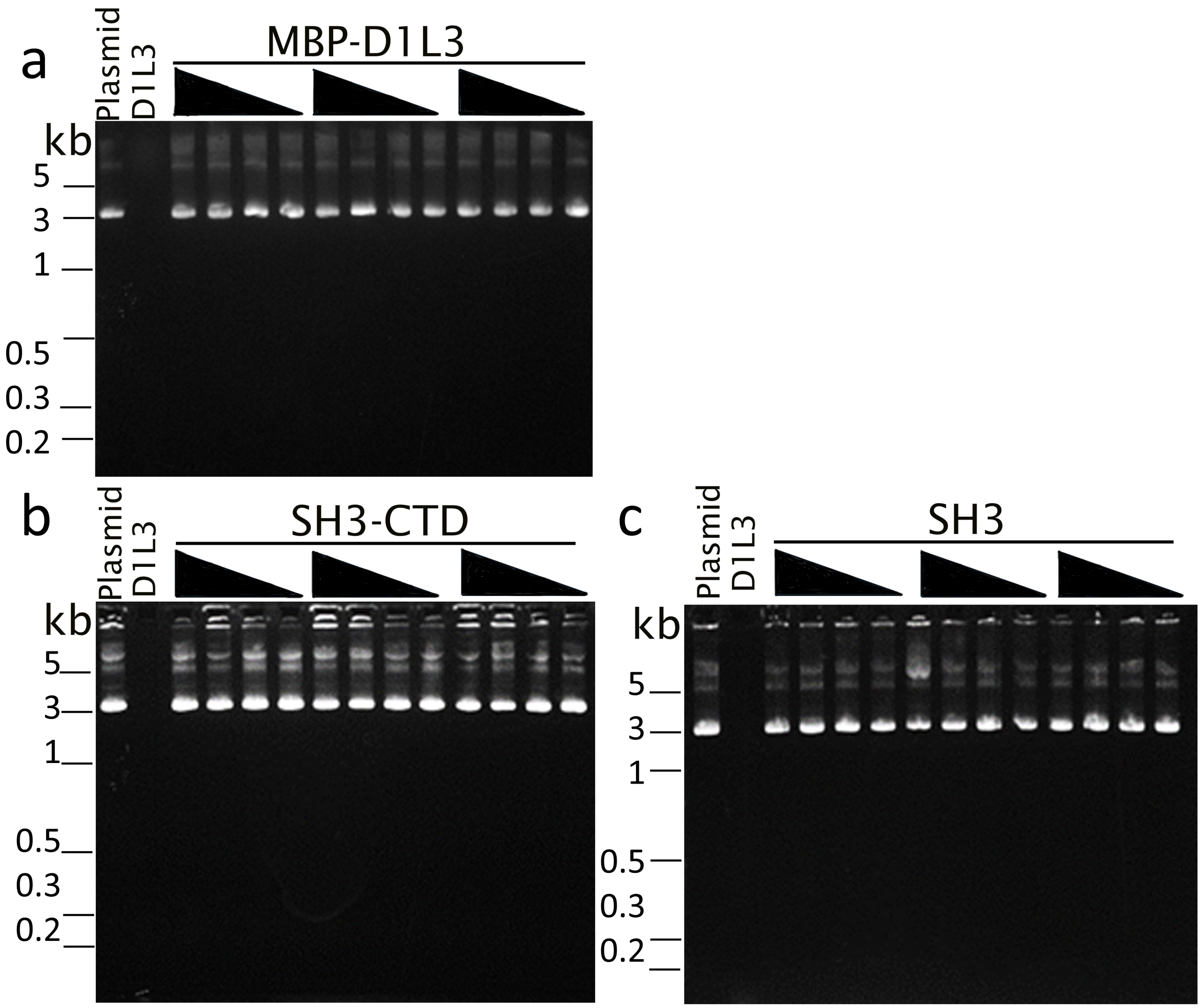


**Figure S1. Plasmid degradation activity for fusion proteins constructs: a)** Plasmid degradation activity of Dnase1L3-MBP fusion protein (D1L3 MBP), **b)** SH3-CTD fusion or **c)** SH3 domain alone was measured by mixing 200 ng of plasmid DNA with a range of concentrations, from 34 ng/$\mu$l to 500 pg/$\mu$l, for 30 min at 37 °C and analyzed on an agarose gel. The first lane in each sample is the DNA-only negative control. The second lane is full-length Dnase1L3 as a positive control.

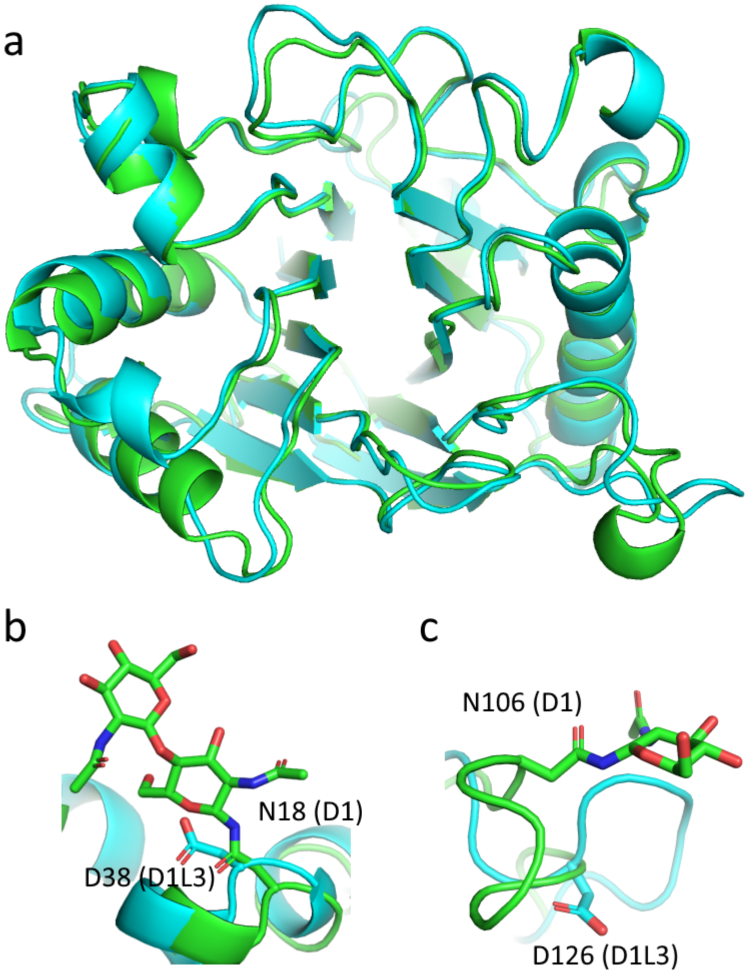


**Figure S2.** **Comparison of the X-ray structures of Dnase1 and the core enzyme of Dnase1L3: a)** The crystal structure of Chain C of Dnase1L3 ΔCTD (PDB:7KIU) in blue aligned to Dnase1 (PDB:4AWN) in green using C-$\alpha$ alignment with an RMSD=1.76 Å. **b)** The homologous residue to the N-linked glycosylation site (Asn-18) in Dnase1 is Asp-38 in Dnase1L3. **c)** The Asn-106 N-linked glycosylation site in Dnase1 corresponds to Asp 126 in Dnase1L3.

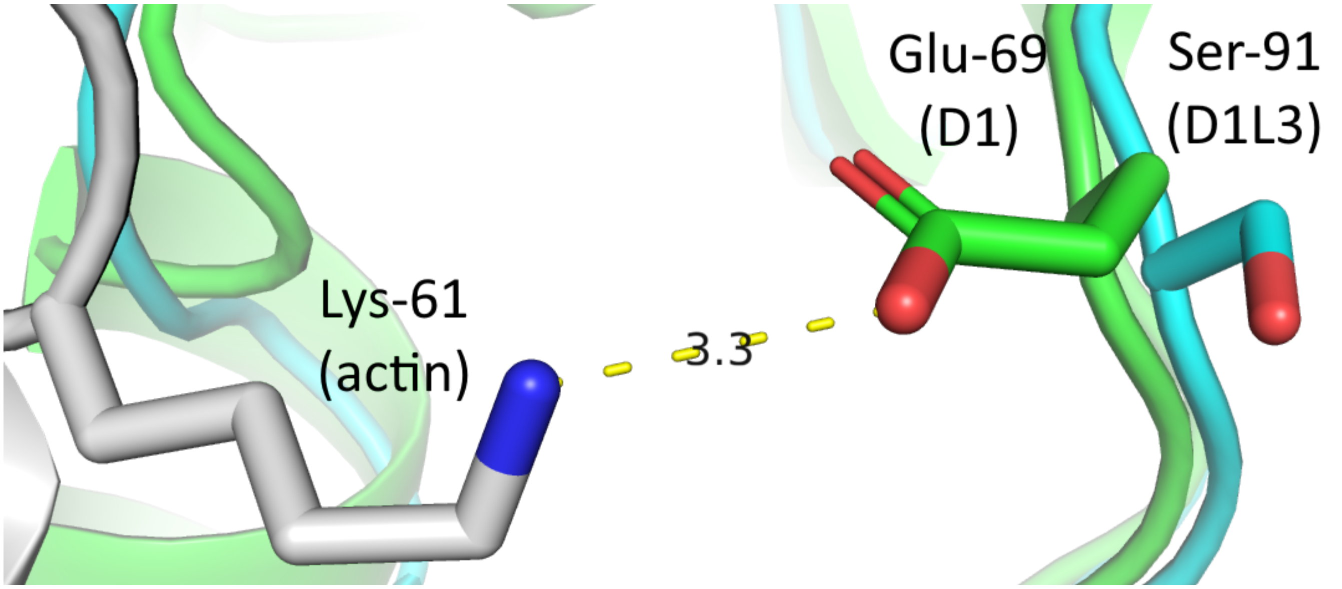


**Figure S3. Disrupted salt bridge contributes to actin resistance in Dnase1L3:** Dnase1 forms a salt bridge to actin between the Dnase1 (D1, green) residue Glu-69 and the actin (grey) residue Lys-61. This salt bridge was shown to be a close contact site by cross-linking experiments and the Dnase1 crystal structure[22, 72]. The corresponding residue to Dnase1 Glu-69 on Dnase1L3 is Ser-91. The calculated distance between a Ser at this locus and the Lys-61 of actin would be > 7 Å. Therefore, it is unlikely that Dnase1L3 could form a productive salt bridge to actin at this locus.

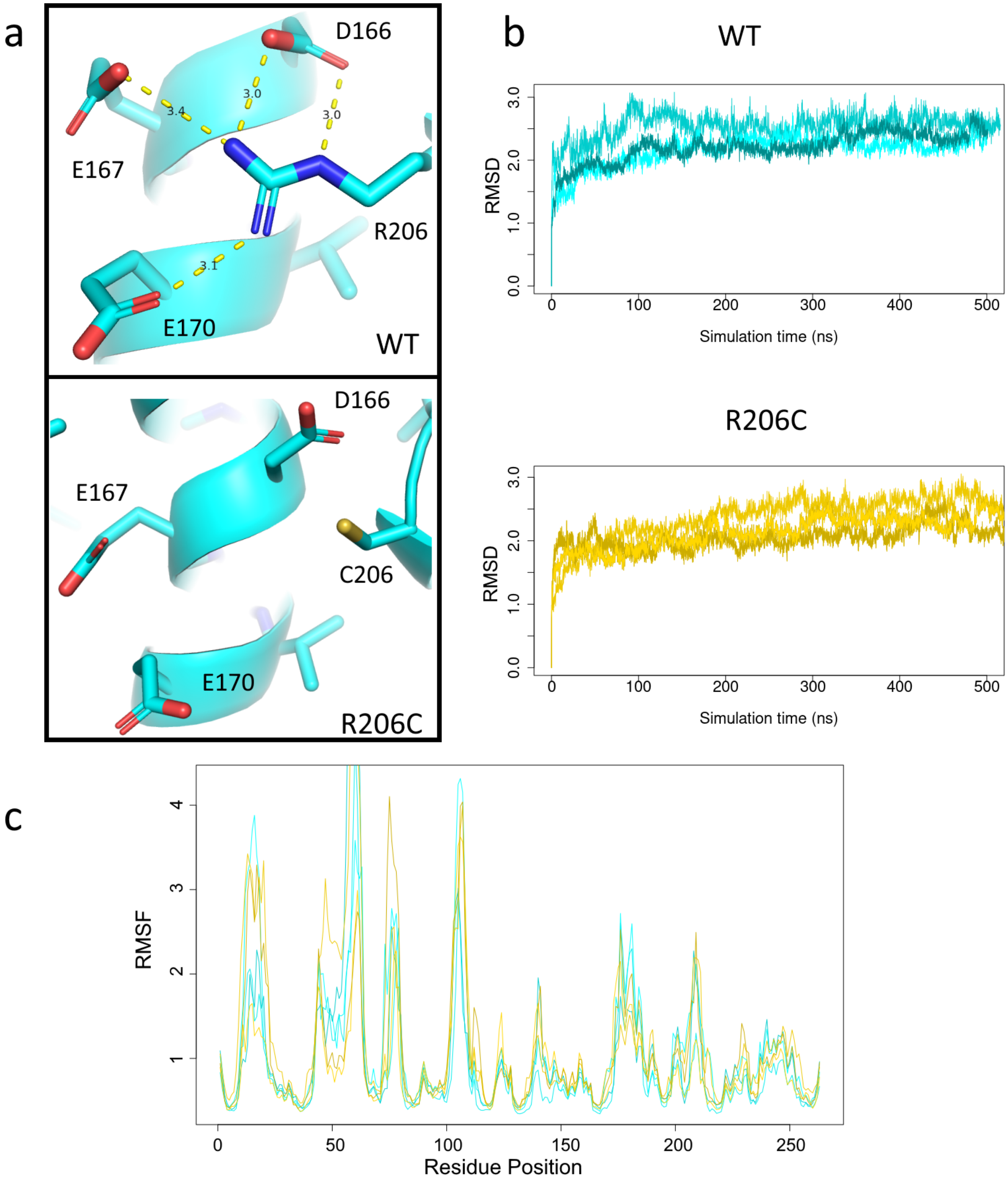


**Figure S4. Molecular Dynamics analysis of the R206C mutation: a)** final snapshot from the 500 ns simulations of either Chain A from the crystal structure of Dnase1L3 with Arg206 (WT) or the R206C mutation. **b)** RMSD from three 500 ns simulations of WT Dnase1L3 (blue) and R206C (yellow). **c)** The RMSF of each of the six MD simulations, WT (Arg-206) is shown in blue and R206C is shown in yellow. There is no significant increase in mobility (RMSF) for the loop residues preceding Arg206, when it is modified to a cysteine residue.

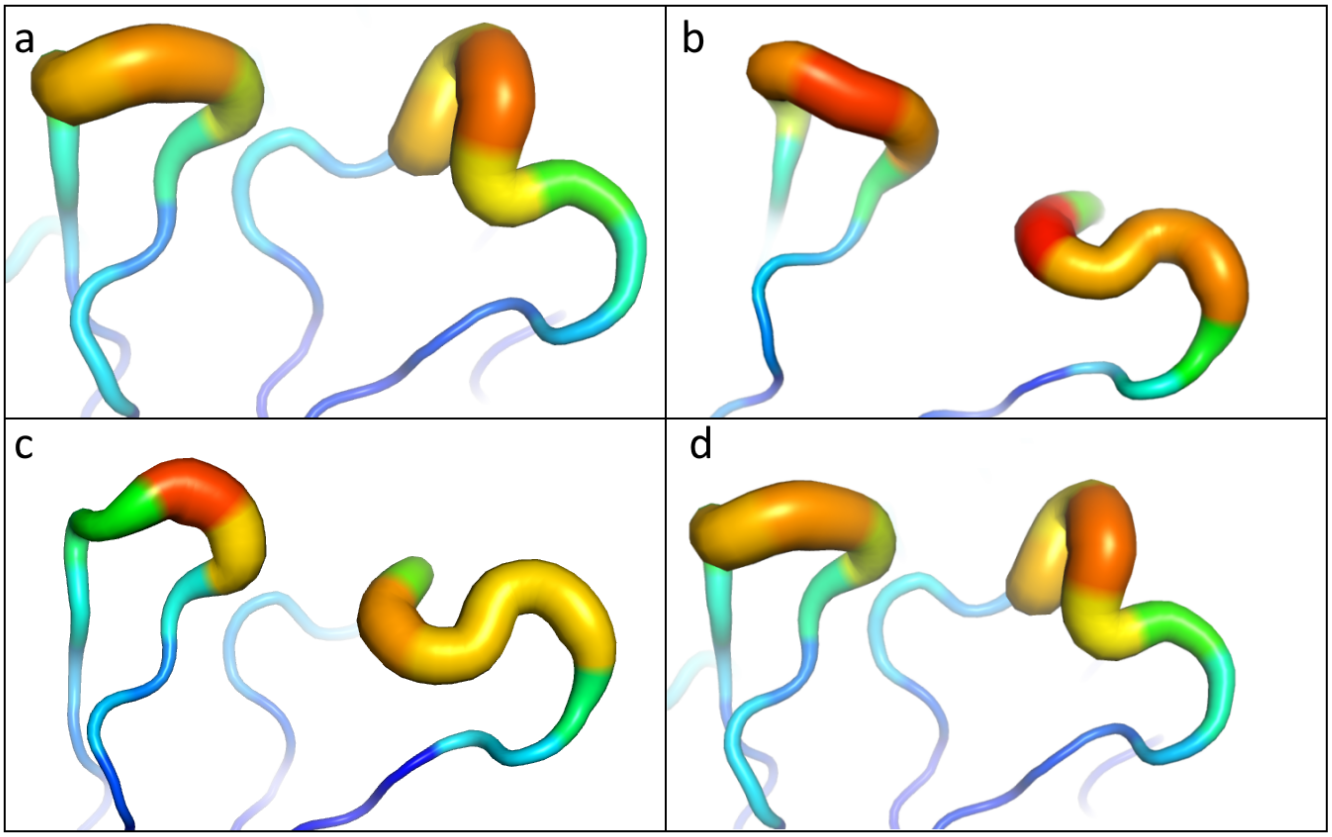


**Figure S5. B-factor putty plot of divalent cation binding site 'II':** Each of the four chains **(a-d)** in the crystallographic asymmetric unit of Dnase1L3 (PDB:7KIU). Blue represents a low-B factor region and red amino acids have a relatively high B-factor region. The highly acidic coordinating residues of the 'II' loop are conserved; however, a missing disulfide bridge in Dnase1L3, compared to Dnase1, disrupts divalent cation coordination. Consistent with a non-coordinating highly charged loop region, the 'II' loop is highly flexible and has increased flexibility as measured by the B-factor putty plot across all four chains.

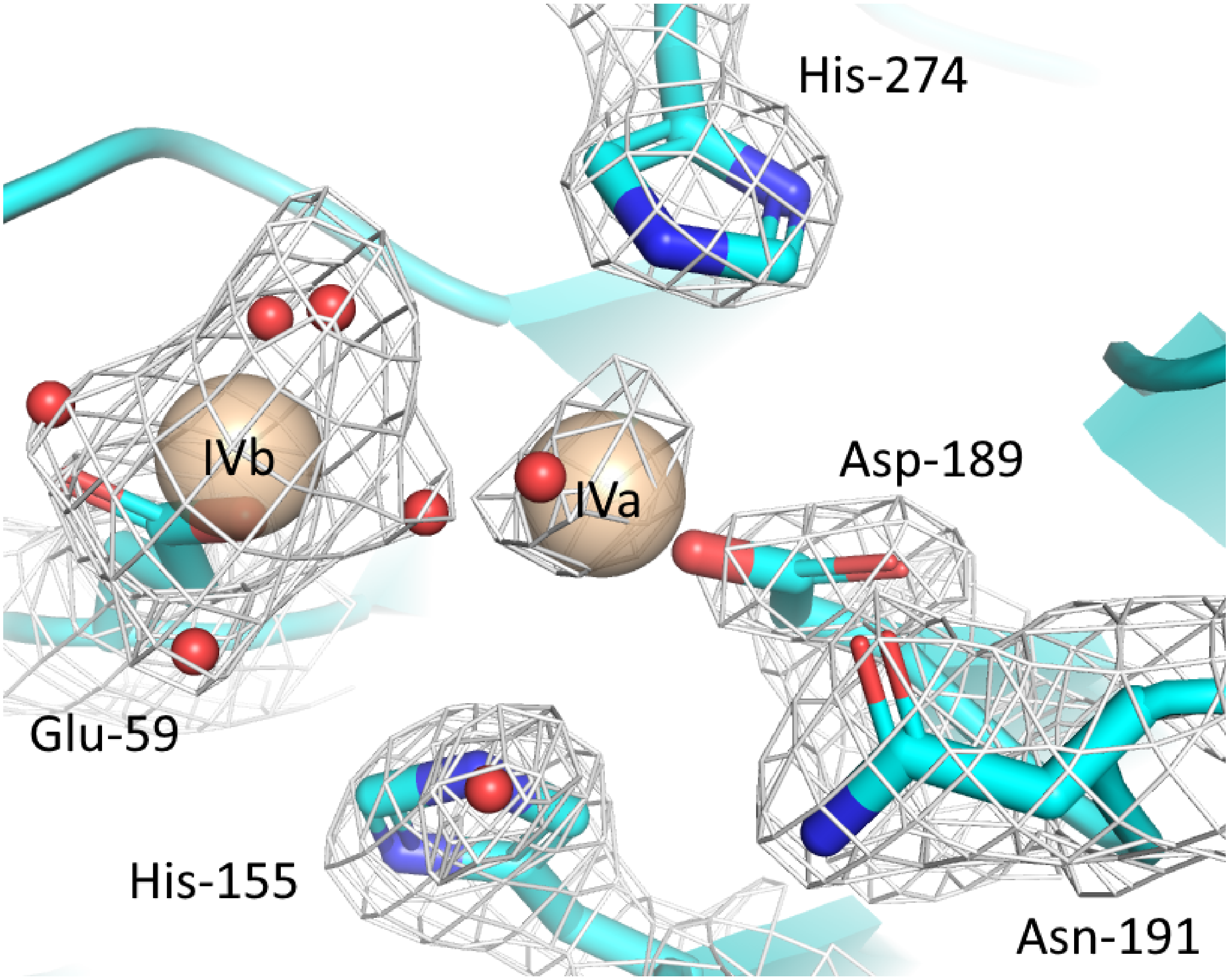


**Figure S6. Dnase1L3 crystallized with two $Mg^{2+}$ in the active site:** The $2F_O$-$F_C$ density is contoured at $1\sigma$. The previously observed 'IVb' divalent cation[10] is coordinated by Glu-59 and five water molecules which are coordinated by Glu-59, Asp-273, and Asn-27. One water molecule is co-coordinated between 'IVb' and 'IVa'. The 'IVa' divalent cation is coordinated by Asp-189, Asn-191, His-274, and three water molecules, one of which is coordinated by His-155 and another is coordinated by Asn-191. The His-274 coordination of 'IVa' was observed to be transient in molecular dynamics simulations. Indeed there is extra density observed between His-274 and 'IVa' that may be an additional coordinating water molecule.

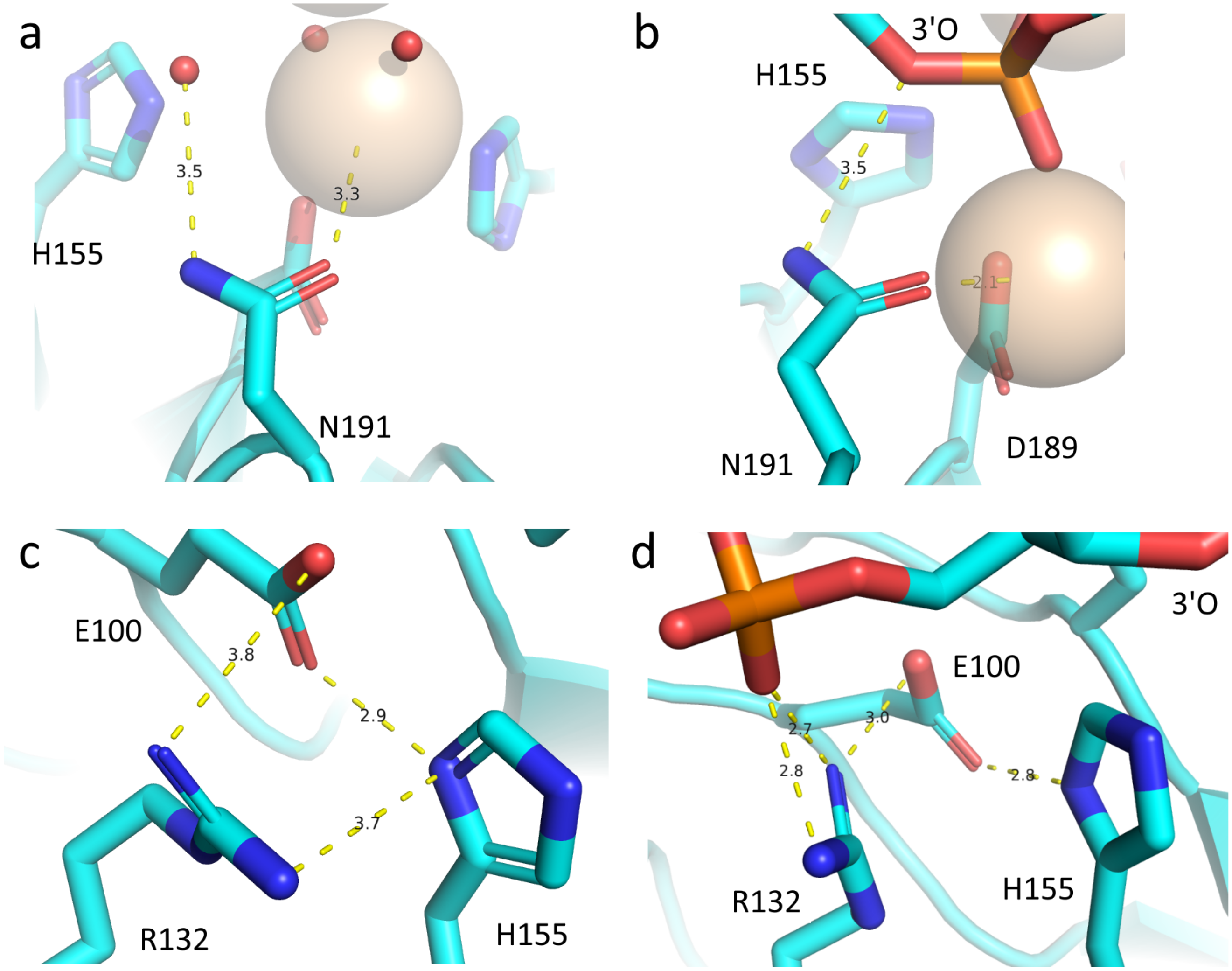


**Figure S7. MD and crystallographic insights into the role of Arg-132 and Asn-191 during catalysis: a)** Arg-132 interacts with His-155 in the Dnase1L3 crystal structure in the absence of DNA: According to our MD simulation, in the presence of DNA **b)** Arg-132 switches to bind the phosphate backbone and Glu-100 alone coordinates His-155. The Arg-132 substrate switch may prime His-155 to accept a proton, thus regenerating the protonating[11] His-155. **c and d)** Asn-191 coordinates the active site $Mg^{2+}$ divalent cation 'IVa' in the Dnase1L3 crystal structure **(c)** and molecular dynamics in the presence of DNA **(d)**. Asn-191 still interacts through the $\delta 2$ amine with the oxygen of a solvent water that roughly corresponds to the location of the 3' oxygen of the scissile phosphate, which is shown coordinated in **(d)**. The red spheres in **(c)** are crystallographic waters and the tan sphere coordinated by N191 in **(c and d)** is the 'IVa' $Mg^{2+}$.

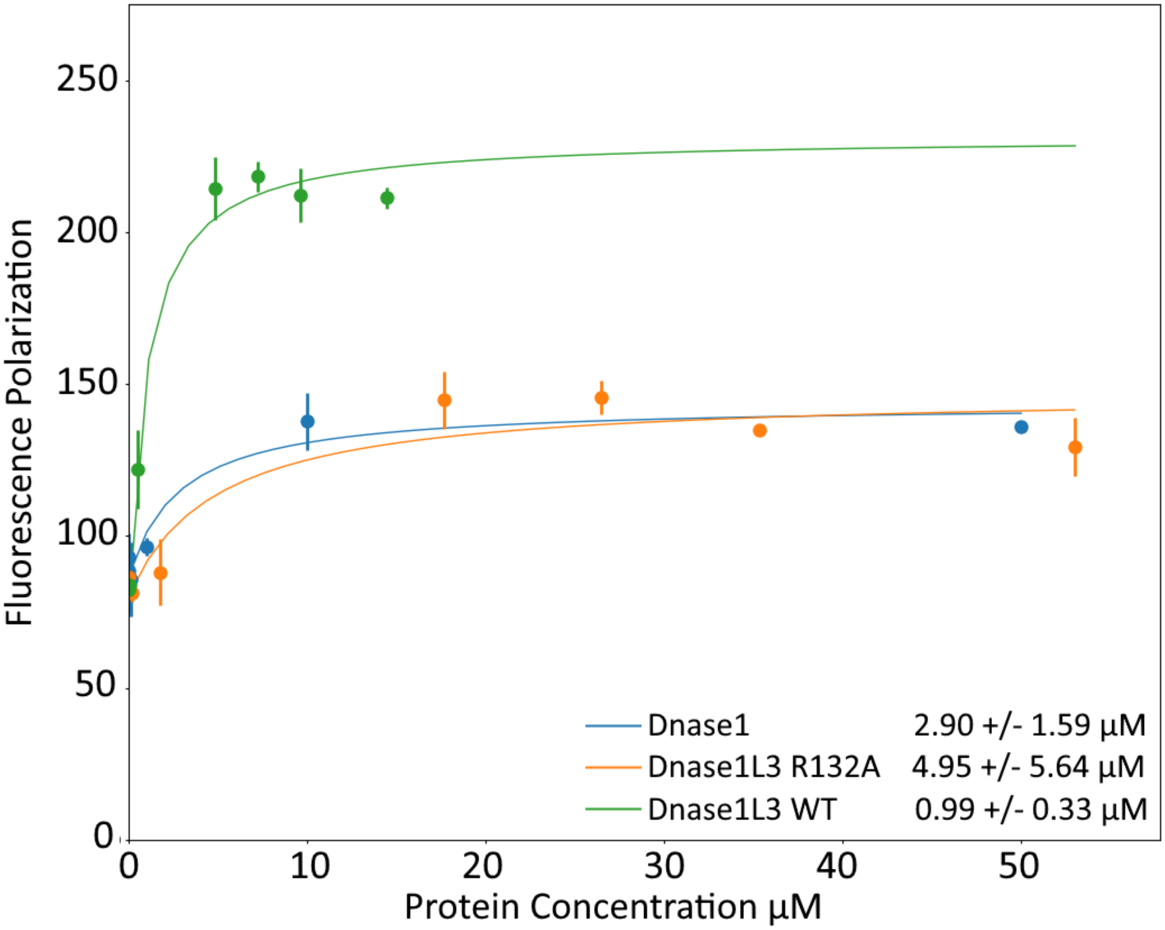


**Figure S8. The R132A mutation decreases DNA affinity:** Dnase1, Dnase1L3 WT, and Dnase1L3 R132A were each incubated with FITC 40-mer dsDNA for 30 min in 1mM EDTA containing binding buffer. The resulting change in fluorescence polarization was indicative of enzyme binding to the DNA substrate. Dnase1L3 WT displayed the highest affinity for dsDNA substrate, 990 nM ± 330 nM. Dnase1 showed a third of the affinity for DNA at 2.9 $\mu$M. Dnase1L3 R132A showed the lowest affinity at 4.95 $\mu$M. The loss in affinity due to the R132A mutation corresponds to 5-fold drop in binding. Indicating that Arg-132 does indeed bind to DNA as predicted by Dnase1 crystal structure, 1DNK, and the molecular dynamics simulations performed based on the Dnase1L3 crystal structure with DNA modelled in the active site. Error bars represent SEM, n=24 for Dnase1L3 WT and R132A and, n=28 for Dnase1, of independent fluorescence polarization recordings for each sample.

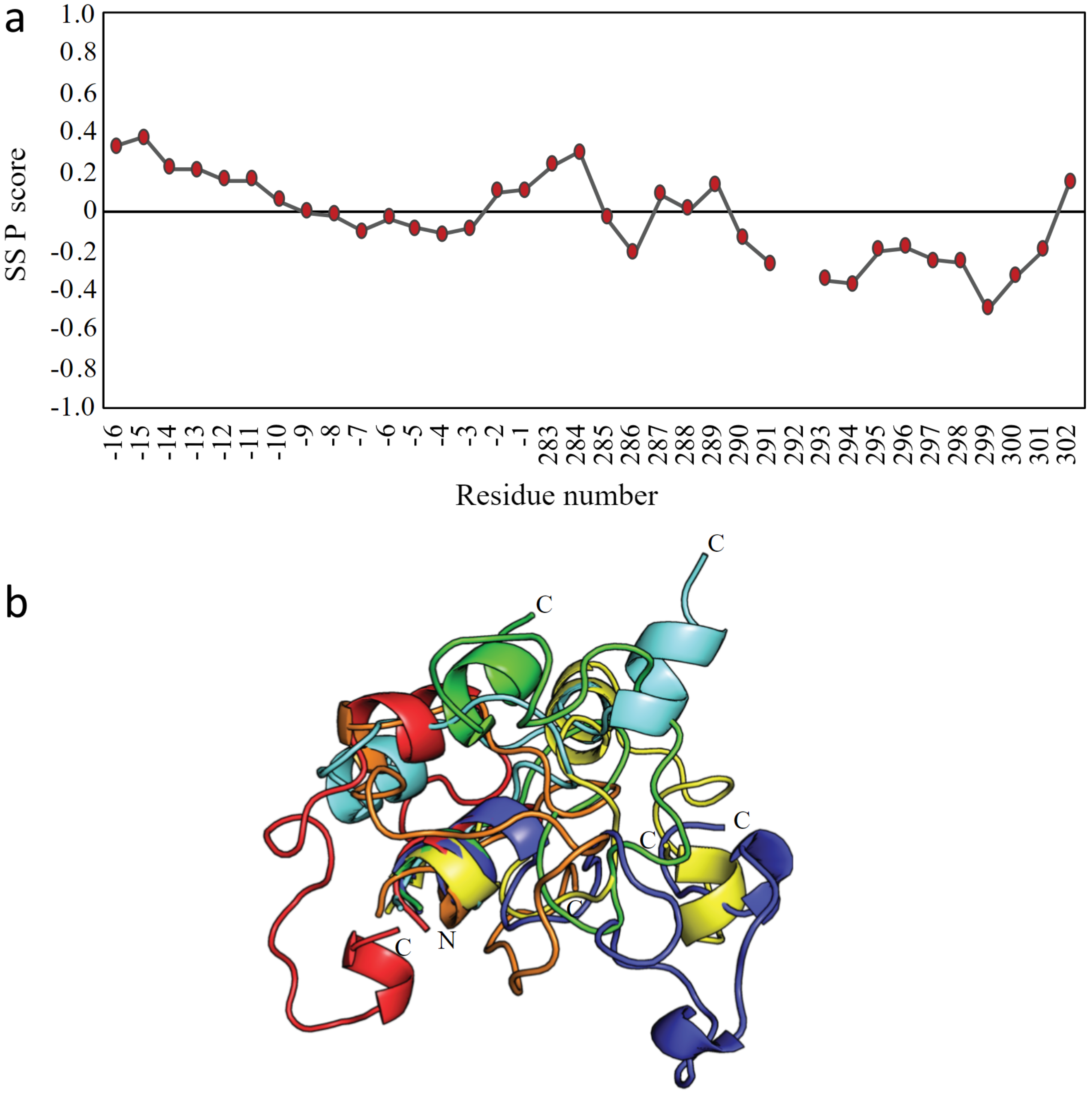


**Figure S9. The CTD of Dnase1L3 is disordered in solution. a)** Secondary structure propensity (SSP) scores for 19 of the amino acids of the CTD of Dnase1L3 (using Dnase1L3 numbering) including the linker with SH3, SGSENLYFQGGSYAMG (numbered -16 to -1), calculated using 13C$\alpha$ and 13C$\beta$ chemical shifts. Positive values represent $\alpha$-helix propensity, negative values represent $\beta$-strand propensity, and values near zero suggest random coil structure[73]. The SSP scores for Lys-292, Lys-303, Arg-304, and Ser-305 are absent due to high uncertainty in the prediction, which is a result of inability to assign the last three amino acids of the CTD (303 to 305) and the C$\beta$ chemical shifts for Lys-292. **b)** Superimposition of the six lowest energy conformers obtained from CS-Rosetta. Structures were aligned to the first ten amino acids.

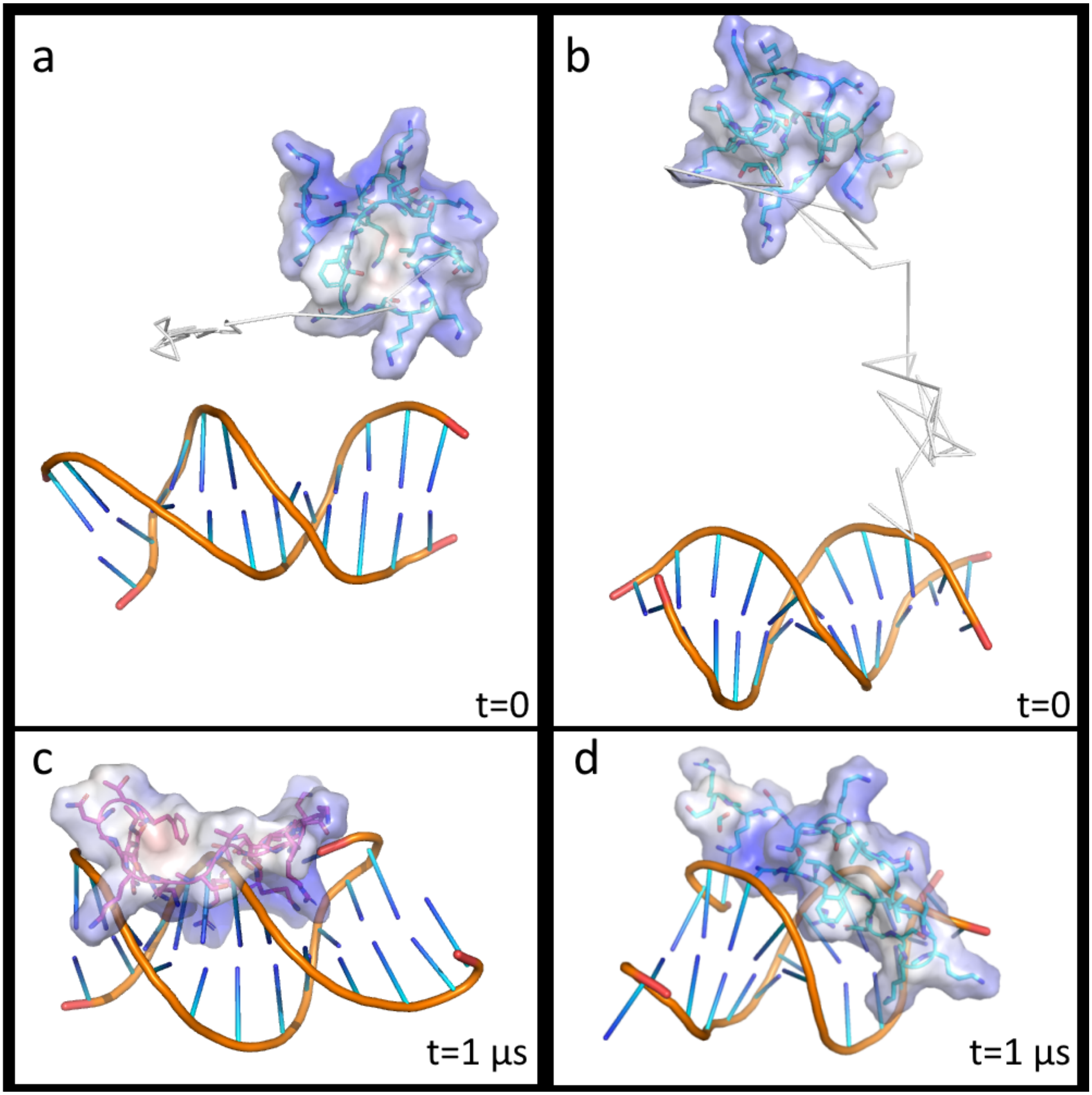


**Figure S10. The CTD structure was resolved with solution-state NMR and analyzed in an explicit solvent box with a 16-mer of dsDNA. a and b)** The initial setup for each simulation is shown. The electrostatic potential of the CTD peptide is indicated by the colored volume surrounding the peptide. Blue shading signifies positively-charged potential. The trajectory was traced over the first 12 ns by plotting the position of a centrally-positioned C-$\alpha$ (Val-294). DNA and the CTD are traced for the first 12 ns to demonstrate that although separated at the start of the simulation, the CTD gravitates immediately towards the 16-mer dsDNA. **c and d)** The final configuration of each CTD-DNA simulation at 1 $\mu$s is shown for the corresponding initial conditions. The CTD does not adopt a distinct secondary structure in the presence of dsDNA, and the two simulations do not converge on a common CTD binding conformation.

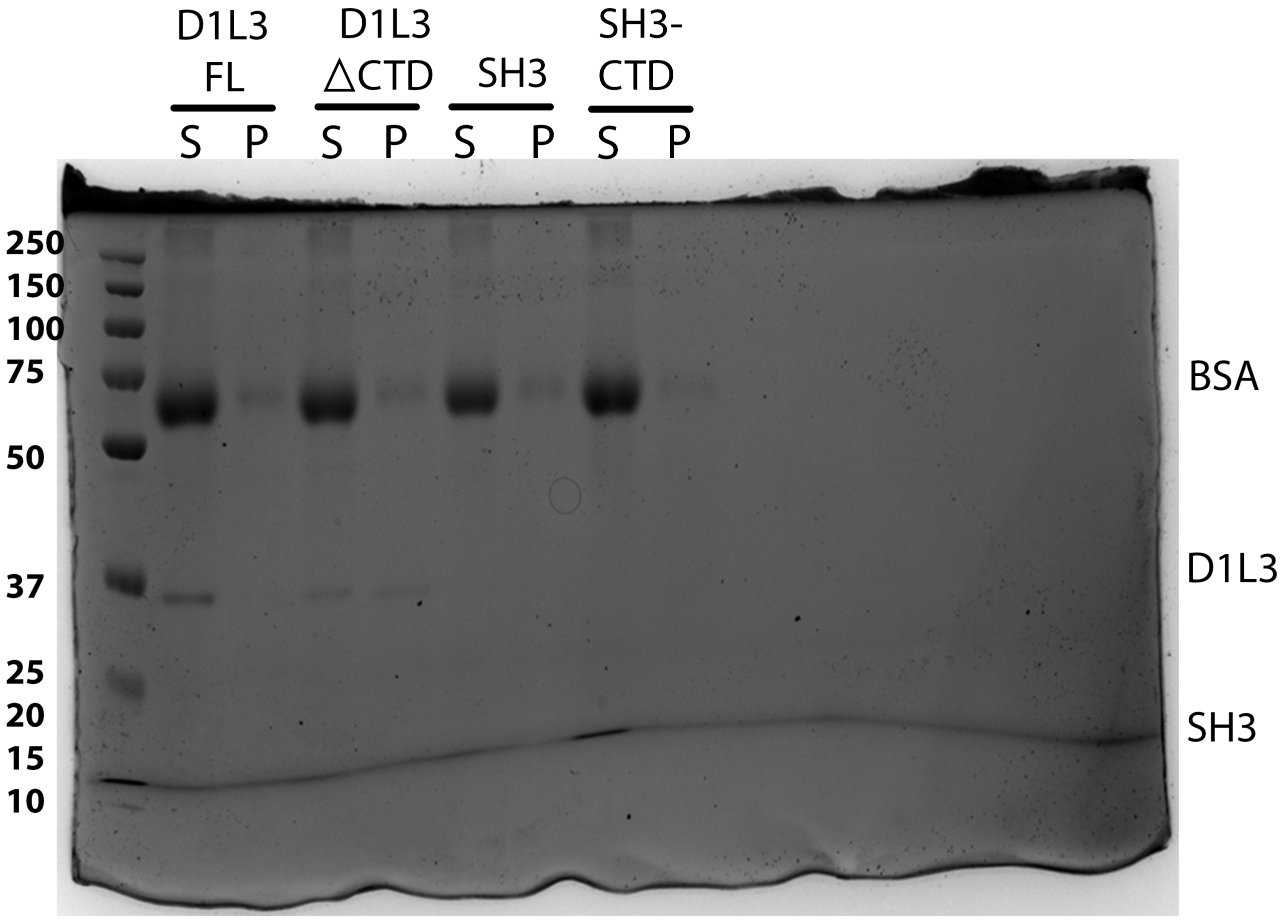


**Figure S11. Uncropped and unedited SDS-PAGE gel from Figure 5e of Liposome binding.** The uncropped and unedited version of the coomassie stained SDS-PAGE gel in Figure 5e. The lane order from left to right is as follows: 1) Ladder 2) Supernatant Dnase1L3 FL 3) Pellet Dnase1L3 FL 4) Supernatant Dnase1L3 ΔCTD 5) Pellet Dnase1L3 ΔCTD 6) Supernatant SH3 7) Pellet SH3 8) Supernatant SH3-CTD 9) Pellet SH3-CTD. BSA represents the darker band between 50 and 75 kDa and Dnase1L3 the lighter band near the 37 kDa lane marker.

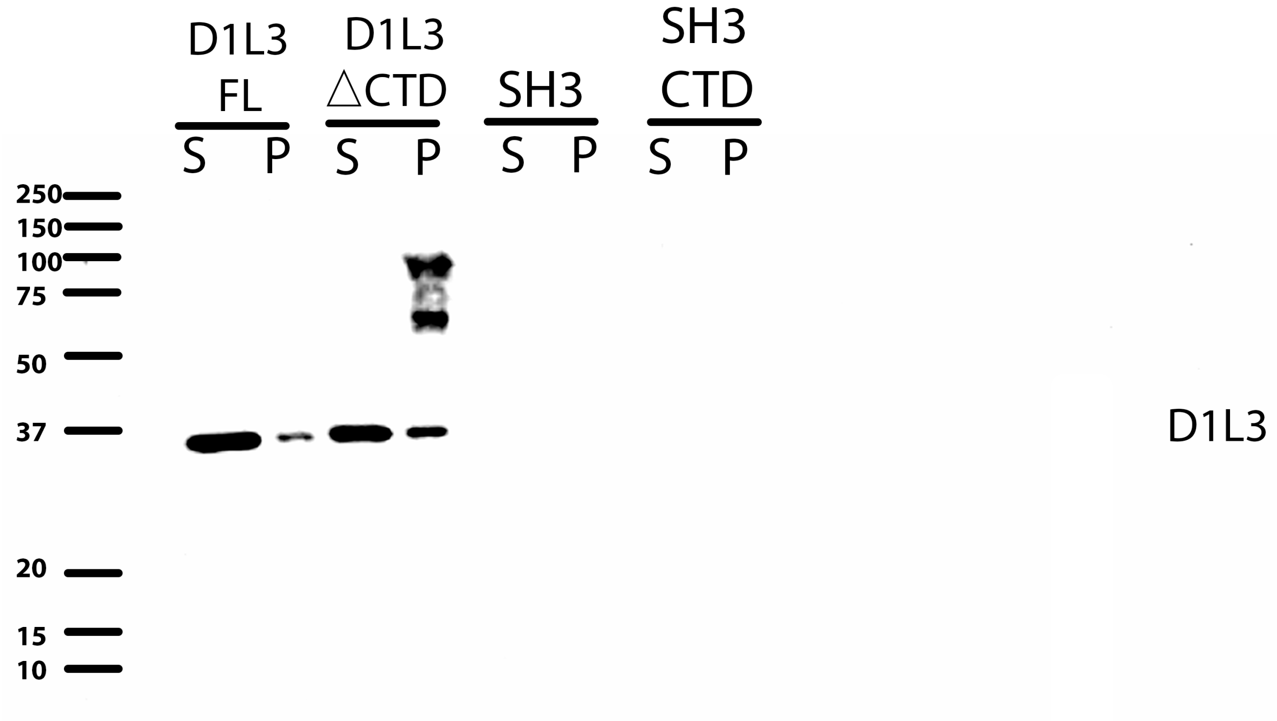


**Figure S12. Uncropped and unedited Immunoblot blot from Figure 5e of Liposome binding.** The uncropped and unedited version of the western blot in Figure 5e. Lane markers are added to the left of the blot based on the SDS-PAGE gel that was transferred the nitrocellulose membrane. The lane order from left to right is as follows: 1) Ladder, 2) Supernatant Dnase1L3 FL, 3) Pellet Dnase1L3 FL, 4) Supernatant Dnase1L3 ΔCTD, 5) Pellet Dnase1L3 ΔCTD, 6) Supernatant SH3, 7) Pellet SH3, 8) Supernatant SH3-CTD, 9) Pellet SH3-CTD. The bands near 250 and 100 kDa in lane 5 represent nonspecific bands.

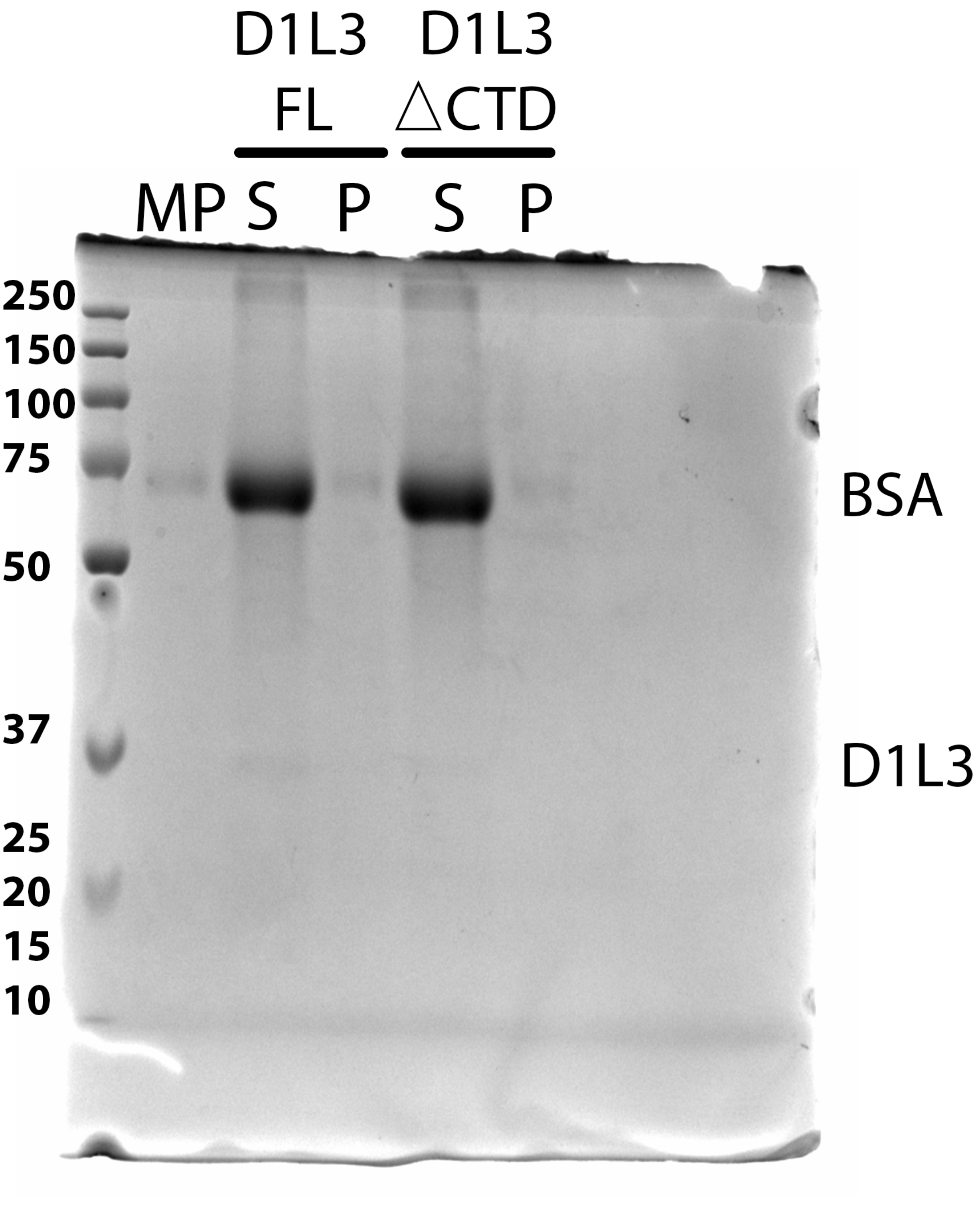


**Figure S13. Uncropped and unedited SDS-PAGE gel from Figure 5f of Microparticle binding.** The uncropped and unedited version of the coomassie stained SDS-PAGE gel in Figure 5f. The lane order from left to right is as follows: 1) Ladder 2) Microparticles alone 3) Supernatant Dnase1L3 FL 4) Pellet Dnase1L3 FL 5) Supernatant Dnase1L3 ΔCTD 6) Pellet Dnase1L3 ΔCTD. BSA represents the darker band between 50 and 75 kDa and Dnase1L3 the lighter band near the 37 kDa lane marker.

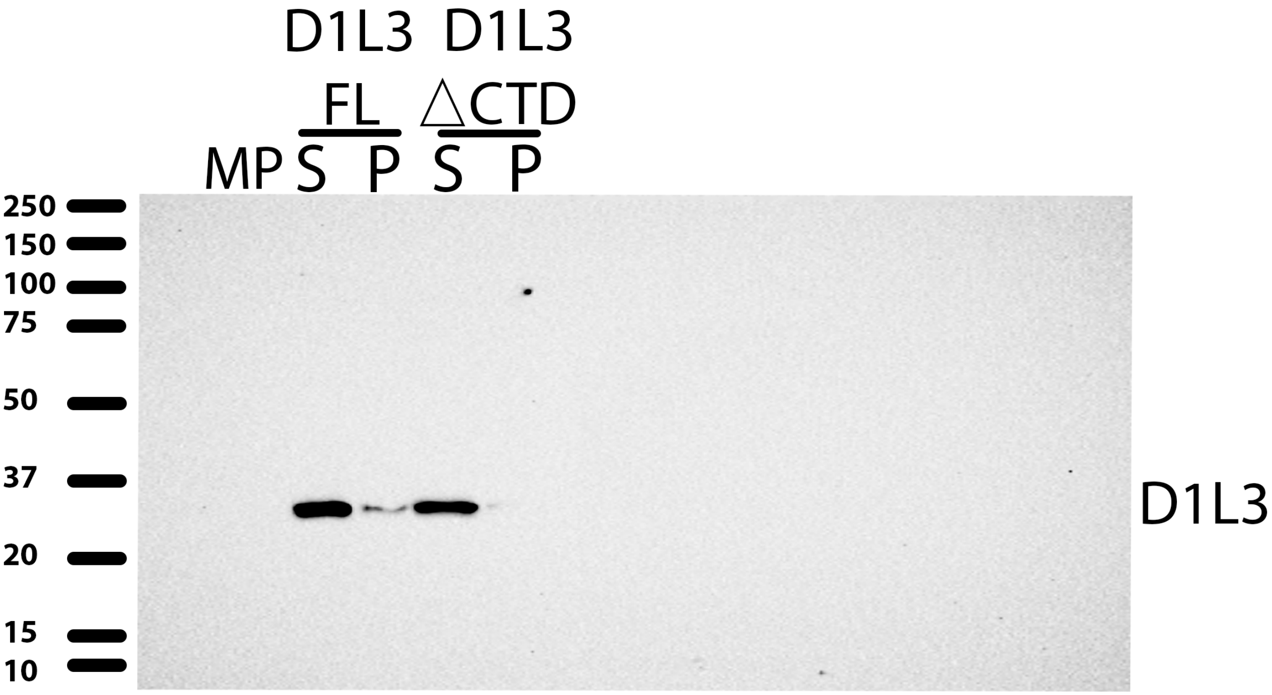


**Figure 14. Uncropped and unedited Immunoblot blot from Figure 5f of Microparticle binding.** The uncropped and unedited version of the western blot in Figure 5f. Lane markers are added to the left of the blot based on the SDS-PAGE gel that was transferred the nitrocellulose membrane. The lane order from left to right is as follows: 1) Microparticles alone 2) Supernatant Dnase1L3 FL 3) Pellet Dnase1L3 FL 4) Supernatant Dnase1L3 ΔCTD 5)Pellet Dnase1L3 ΔCTD.
