# Supplementary material for: Structural features of Dnase1L3 responsible for serum antigen clearance
Source: Commun Biol. 2022 Aug 16;5:825. doi: 10.1038/s42003-022-03755-5 (PMC9381713; doi:10.1038/s42003-022-03755-5)
Supplement: Supplementary file 3 — Description of Additional Supplementary Files [file 42003_2022_3755_MOESM3_ESM.pdf]

Description of Additional Supplementary Files

File name: Supplementary Data 1
Description: : Data points for Supplementary Figure S4, panel c – RMSF plot. XLSX format.

File name: Supplementary Data 2
Description: Data points for Supplementary Figure S4, panel b – RMSD plot. XLSX format.

File name: Supplementary Data 3
Description: Data points for the ANS fluorescence in Figure 2 b. XLSX format.

File name: Supplementary Data 4
Description: Dataframe for Figure 2c, Estimated ΔG of WT and R206C. XLXS format.

File name: Supplementary Data 5
Description: : Data points for Figure 4d. Active site calculated pKa values. Format XLXS.

File name: Supplementary Data 6
Description: Data for fluorescence Polarization. Format XLXS.

File name: Supplementary Data 7
Description: Data for Figure 5a. EC50. Format XLXS.

File name: Supplementary Data 8
Description: Data for Figure 5b. Barrier to transfection data. Format XLXS.

File name: Supplementary Data 9
Description: Data for Figure 5c. Immune Complex Degradation. Format XLXS.

File name: Supplementary Data 10
Description: Data for Figure 5d. EC50 for immune complex degradation. Format XLXS.

File name: Supplementary Data 11
Description: Data for Figure 6a. Fluorescence Polarization of DNA + CTD. Formation XLXS.

File name: Supplementary Data 12
Description: Excel Workbook of Figure 6c, circular dichroism data at various [DNA]. Format XLXS.

File name: Supplementary Data 13
Description: Excel Workbook of SAXS data for Figure 6e. Format XLXS.
